# Digitalized therapy and the unresolved gap between artificial and human empathy

**DOI:** 10.3389/fpsyt.2024.1522915

**Published:** 2025-01-07

**Authors:** Roshini Salil, Binny Jose, Jaya Cherian, Sheeja P. R, Nisha Vikraman

**Affiliations:** ^1^ Mount Carmel College of Teacher Education, Kottayam, India; ^2^ Department of Health and Wellness, Marian College Kuttikkanam Autonomous, Kuttikkanam, India; ^3^ Department of Social Work, Vimala College, Thrissur, India; ^4^ Department of Home Science, HHMSPB NSS College for Women, Neeramankara, Thiruvananthapuram, India; ^5^ Department of Home Science, St.Teresa’s College (Autonomous), Ernakulam, India

**Keywords:** artificial empathy, AI in psychotherapy, therapeutic mechanisms, emotion recognition, hybrid therapy models

## Introduction

Empathy is a cornerstone in psychotherapy for building trust, connection, and understanding between therapist and client. Studies and meta-analyses continue to support that therapist empathy significantly correlates with positive therapeutic outcomes (1–3). However, empathy is not the sole pathway to psychological change. Constructs such as validation, autonomy support, attunement, and authentic curiosity also contribute significantly to recovery and mental well-being (4). There are recent development in importance of some non-interpersonal methods, including training in mindfulness, expressive writing, training in focusing, and computer-aided cognitive bias modification; these, too have produced psychological changes with favorable outcome. (5).

Given this multi-psychological framework, how essential empathy is as a core construct from which psychological interventions take part remains a moot debate. The role of empathy in psychotherapy is powerful and influential but only part of the whole net of therapeutic mechanisms (6). This paper discusses the special significance of empathy in psychological change, its limitations, and the risks associated with misrepresentations by AI. It postulates that AI’s strengths may be better utilized in the enhancement of non-empathic therapeutic pathways and hence provides an alternative focus for AI in mental health care.

## Empathy and pathways to psychological change

Empathy has traditionally been regarded as the backbone of the therapeutic relationship. It is a multicomponent concept involving emotional resonance or sharing feelings, cognitive perspective-taking or understanding another’s viewpoint, and compassionate action or taking steps to alleviate distress (7). These dimensions enable a therapist to offer an environment that is non-critical and safe. However, there is an emerging body of research that questions whether empathy provides the sole determinant of psychological change. Instead, other therapeutic factors may be equally, if not more, important (5, 8). For example, validation confirms the client’s feelings and experiences in an effort to establish a sense of trust and reduce feelings of isolation. Similarly, promoting autonomy support-for instance, encouraging clients to take responsibility for their own healing process-promotes long-term recovery and also aligns with modern therapeutic models that are centered around the client (9). The therapist’s attunement-approach, in which he aligns himself with the client’s emotional state, improves rapport. The curiosity of the therapist is conveyed by interest and exploratory questions that promote self-reflection and insight (10, 11).

Beyond interpersonal mechanisms, there are also some very important empathy-free interventions for psychological change. An example is mindfulness-based interventions: MBSR has proved successful in reducing stress and enhancing the regulation of mood (12). Experiences of expressive writing about emotional events enable insight and active emotional processing for better mental health consequences (13). On the other hand, cognitive bias training even spots and corrects negative thinking so as to address the symptoms of anxiety and depression (14). Gendlin’s focusing training, which emphasizes the role of body awareness in emotional processing, has also been tested and found to be an effective therapeutic intervention (15). These interpersonal and non-interpersonal mechanisms underpin, together, the multifaceted nature of psychological change and emphasize how AI needs to augment rather than try to replace such pathways.

## The shortfall of artificial empathy

Artificial empathy is a feature of AI, whereby it is able to recognize and then simulate empathic responses based on data such as text, tone, and facial expressions (16). While indeed a great achievement in technology, AI lacks depth, intentionality, and cultural sensitivity, which are very important ingredients for emotional resonance (17, 18). These limitations of AI appear most manifestly in three areas: First, it struggles with contextual understanding since it cannot construct a holistic understanding of an individual’s life experiences, because it is unable to recognize emotional meaning in context, that would be the first limit it faces. The second one is cultural insensitivity, as algorithms of emotion recognition in AI are quick to misinterpret or simplify emotional cues across different cultural contexts. Finally, AI lacks emotional resonance, in that it cannot draw from lived experiences in service of deeper connections with clients. These limitations emphasize the risks of relying on AI in emulating empathy in mental health care.

Despite these setbacks, it is argued that through the simulation of empathy, AI democratizes mental health care insofar as it increases access to services (19). AI systems can provide immediate support and serve as entry points for those who may feel uneasy with traditional therapy (20). Poorly aligned or over-and-over robotic responses could alienate clients and destroy any trust that might be needed for a successful therapeutic relationship (21). Given these risks, perhaps AI’s role should shift to support other therapeutic mechanisms rather than trying to emulate empathy by, for example, training on giving real-time, emotional feedback or generating personalized insights to support human therapists.

## Charting the path forward: future research in AI-guided therapy

Future research priorities must be addressed in securing a position whereby AI is developing responsible and effective mental health. Development of multi-modal emotion recognition systems that combine evidence from text, speech, facial expressions, and physiological signals would be an essential approach to go about understanding emotions holistically (22). With long-term effectiveness, an empirical study on hybrid therapy models by AI and a human should be assessed regarding impacts on the outcomes and satisfaction of clients. This will also be immensely useful in trusting and informing user-friendly system designs while understanding the perceptions of clients about AI in therapy. Furthermore, for the refinement of the ethical guidelines, there is a requirement to address challenges in data privacy, transparency, and consent (23). Lastly, other roles that could well be investigated in AI include monitoring client progress, personalization of treatment plans, and supporting non-interpersonal therapeutic pathways such as mindfulness and expressive writing that have the potential to extend its utility while minimizing risks.

## Conclusion

While empathy is essential to psychotherapy, it is neither the sole nor irreplaceable pathway to psychological change. Evidence underlines alternative mechanisms that are effective, including validation, autonomy support, and mindfulness training. Given the risk of AI misinterpreting empathy, its role should be focused on enhancing non-empathic therapeutic factors and supporting hybrid therapy models. Coupled with research and ethical developments, AI has the potential to enhance mental health treatment delivery without losing its human-centered approach. In the future, it is envisaged that AI will act not as a substitute but as an influential ally in solving the rapidly increasing demand for mental health services.
